# Structural Basis of Native CXCL7 Monomer Binding to CXCR2 Receptor N-Domain and Glycosaminoglycan Heparin

**DOI:** 10.3390/ijms18030508

**Published:** 2017-02-26

**Authors:** Aaron J. Brown, Krishna Mohan Sepuru, Krishna Rajarathnam

**Affiliations:** Department of Biochemistry and Molecular Biology, and Sealy Center for Structural Biology and Molecular Biophysics, The University of Texas Medical Branch, Galveston, TX 77555, USA; aj3brown@utmb.edu (A.J.B.); kmsepuru@utmb.edu (K.M.S.)

**Keywords:** chemokine, CXCL7, NAP-2, CXCR2, glycosaminoglycan, heparin, NMR, monomer

## Abstract

CXCL7, a chemokine highly expressed in platelets, orchestrates neutrophil recruitment during thrombosis and related pathophysiological processes by interacting with CXCR2 receptor and sulfated glycosaminoglycans (GAG). CXCL7 exists as monomers and dimers, and dimerization (~50 μM) and CXCR2 binding (~10 nM) constants indicate that CXCL7 is a potent agonist as a monomer. Currently, nothing is known regarding the structural basis by which receptor and GAG interactions mediate CXCL7 function. Using solution nuclear magnetic resonance (NMR) spectroscopy, we characterized the binding of CXCL7 monomer to the CXCR2 N-terminal domain (CXCR2Nd) that constitutes a critical docking site and to GAG heparin. We found that CXCR2Nd binds a hydrophobic groove and that ionic interactions also play a role in mediating binding. Heparin binds a set of contiguous basic residues indicating a prominent role for ionic interactions. Modeling studies reveal that the binding interface is dynamic and that GAG adopts different binding geometries. Most importantly, several residues involved in GAG binding are also involved in receptor interactions, suggesting that GAG-bound monomer cannot activate the receptor. Further, this is the first study that describes the structural basis of receptor and GAG interactions of a native monomer of the neutrophil-activating chemokine family.

## 1. Introduction

**Figure 1:**
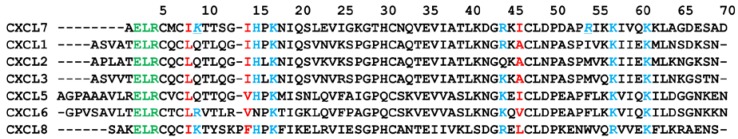
Sequence alignment of neutrophil-activating chemokines. The conserved “ELR” motif is shown in green. Basic residues that mediate GAG and receptor interactions and hydrophobic residues that mediate receptor interactions for CXCL7 identified in this study are shown in blue and red, respectively. The corresponding residues in other chemokines are likewise highlighted. Residues K9 and R54 shown to be involved in binding only in CXCL7 are italicized and underlined.

Chemokines, a large family of signaling proteins, mediate diverse biological functions, including inflammation, development and tissue repair [1,2,3]. Chemokines mediate their function by activating seven transmembrane G-protein coupled receptors (GPCRs) and binding sulfated glycosaminoglycans (GAGs) that regulate receptor function [4,5,6]. Another key feature of chemokines is their ability to reversibly exist as monomers and dimers and sometimes as higher order oligomers. Humans express ~50 different chemokines, which are classified on the basis of conserved cysteines near the N-terminus as CXC, CC, CX_3_C and XC. Chemokine CXCL7 (also known as NAP-2), released by activated platelets, plays a prominent role in recruiting neutrophils to the injury site during thrombosis [7,8,9]. CXCL7 belongs to a subset of CXC neutrophil-activating chemokines (NACs) that are characterized by an N-terminal “ELR” motif and function as potent agonists for the CXCR2 receptor [10]. Other members of ELR-chemokines include CXCL1, CXCL2, CXCL3, CXCL5, CXCL6 and CXCL8 (Figure 1).

Monomer-dimer equilibrium constants have been determined for CXCL1, CXCL5, CXCL7 and CXCL8. Among them, CXCL7 stands out, as it forms a much weaker dimer [11,12,13,14]. Whereas the dimerization constant for CXCL7 is ~50 to 100 μM, the values for other chemokines vary around ~1 to 10 μM. We observed that the dimer levels increase with increasing concentration up to a point after which tetramer levels populate, and dimer levels do not go beyond ~50% at any given condition (Table S1). The structure of CXCL7 determined by crystallography corresponded to the tetrameric state [15], which is not surprising, as crystallography by its very nature results in the higher oligomeric state. The solution structure of a CXCL7 monomer determined in the presence of 2-chloroethanol that is known to disrupt intermolecular dimer and tetramer interactions has been reported, but its coordinates are not available in the public domain [16].

Receptor binding and activity measurements have shown that CXCL7 binds CXCR2 with nanomolar (nM) affinity, indicating that the monomer is a potent agonist [17]. Presently, nothing is known regarding the structural basis or molecular mechanisms by which the CXCL7 monomer interacts with the receptor. Knowledge of the GAG interactions is also essential as GAG interactions regulate receptor function. Using solution nuclear magnetic resonance (NMR) spectroscopy, we characterized the binding of the native CXCL7 monomer to the CXCR2 N-terminal domain (N-domain) that functions as a critical ligand binding site and heparin that serves as a representative and well-studied, sulfated GAG. Towards this end, we first assigned the chemical shifts of the native monomer that are essential for characterizing receptor and GAG interactions and also developed a chemical shift-based structural model of the CXCL7 monomer. We observed that receptor binding is largely mediated by hydrophobic interactions, that electrostatic and H-bonding interactions also play a role and that the CXCR2 N-domain binds a groove comprising the N-loop and adjacent β-strand residues. On the other hand, heparin binding is predominantly mediated by electrostatic interactions. We also observe that heparin adopts different binding geometries and that the binding interface is highly plastic. Most interestingly, our data indicate that GAG-bound CXCL7 monomer cannot bind the receptor. Further, to our knowledge, this is the first study characterizing the GAG and receptor interactions of a native neutrophil activating chemokine monomer.

## 2. Results

### 2.1. CXCL7 Monomer Chemical Shift Assignments

**Figure 2:**
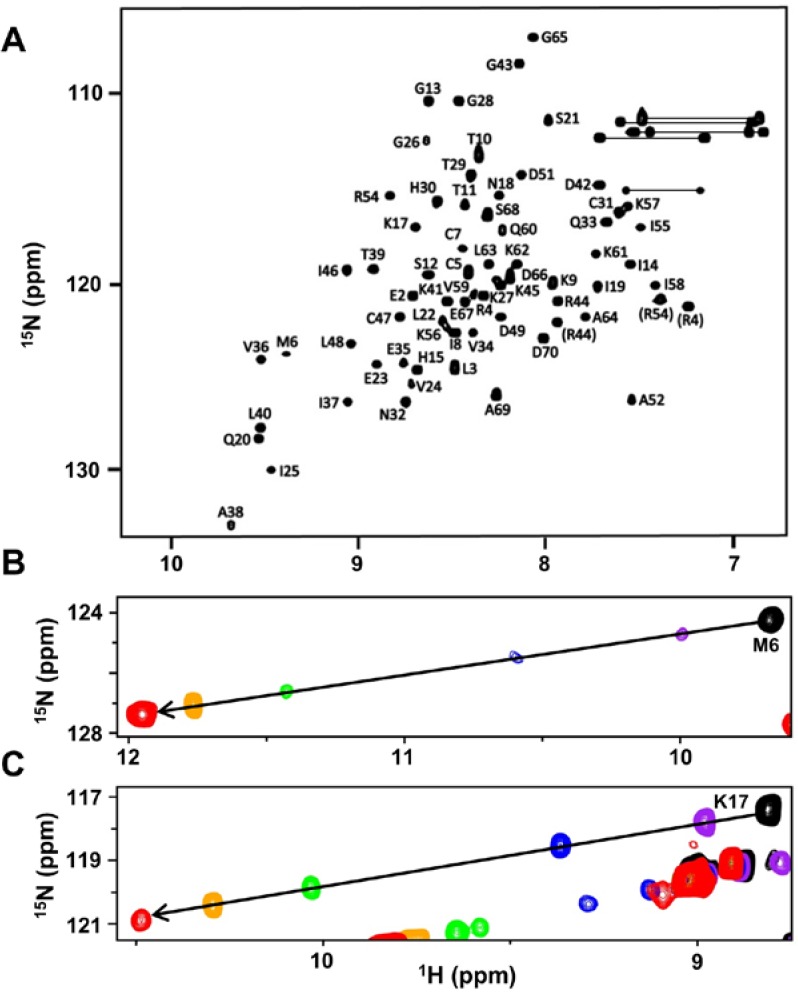
NMR characteristics of the CXCL7 monomer. (**A**) The HSQC spectrum shows excellent chemical shift dispersion indicating a well-folded single species at pH 4.0. The folded arginine side chain peaks are indicated in closed brackets; (**B**,**C**) Large chemical shift changes observed as a function of pH are shown for M6 and K17. The transition is from pH 4.2 (black), 4.4 (purple), 5.0 (blue), 5.5 (green) 6.0 (orange) to 7.0 (red).

Chemical shifts are exquisitely sensitive to local changes in the electronic environment and, as such, serve as useful probes for mapping the binding interface of macromolecular interactions. The binding interface is inferred from binding-induced chemical shifts obtained from heteronuclear single quantum coherence (HSQC) titrations of an unlabeled ligand to a ^15^N-labeled protein. Therefore, knowledge of the native CXCL7 monomer chemical shifts is essential to describe the molecular basis of receptor and GAG interactions. We first characterized how monomer/dimer/tetramer levels vary as a function of buffer, temperature and pH from relative peak intensities in the 2D HSQC spectra. A summary of the distribution is shown in Table S1. Our data indicate that pH had the highest impact, with the monomer dominating at lower pH and tetramer dominating at higher pH. The dimer was always observed in the presence of monomer or tetramer and was not prevalent at any pH. Other variables such as temperature, ionic strength and buffer condition had much less effect. On the basis of these experiments, we settled on a 300-µM sample at pH 4.0 for monomer assignments. Under these experimental conditions, the protein exists as 95% monomer with the remaining 5% as dimer. The HSQC spectrum under these conditions is shown in Figure 2A. A table of the chemical shifts is also shown (Table S2). As we carried out receptor and GAG interactions at pH ≥6 that better reflects physiological conditions, chemical shifts at these pH are needed. We recorded HSQC spectra as a function of pH from 4.0 to 7.5 that allowed assigning monomer chemical shifts at the higher pH despite elevated dimer levels (Figure S1). The backbone assignments at pH 6.0 were also confirmed using triple resonance experiments. Interestingly, HSQC spectra collected as a function of pH also identified several intramolecular interactions. The M6 amide proton is significantly downfield shifted at higher pH (Figure 2B), and the corresponding residue in various human and murine chemokines is also downfield shifted [13,18,19,20,21,22,23]. On the basis of previous mutagenesis studies in CXCL8 and CXCL1, the chemical shift profile of M6 can be attributed to an intramolecular H-bond between the E35 side chain carboxylate and M6 amide proton [24]. The K17 amide proton is likewise downfield shifted (Figure 2C), which can be attributed to H-bonding to the imidazole group of H15 [25]. The corresponding residue in other NACs has also been shown to be downfield shifted. Mutagenesis studies in related NACs have also shown that these interactions are critical for receptor function [26,27]. Further, we also observed significant chemical shift changes for C7, and most interestingly, two distinct peaks at pH 5.0 and a distinct shoulder could also be observed at higher pH. Structures have shown that the disulfides are dynamic and that the disulfides, in addition to structure, also play crucial roles in receptor function [28].

### 2.2. Structural Model of the Native Monomer

A structure of the ethanol-induced monomer of CXCL7 has been previously reported, but its coordinates are not available in the protein data bank [16]. Generally, nuclear Overhauser effect (NOE)-driven structures require a sufficient number of long-range NOEs to describe different structural elements, their relative orientation and the global fold. We could not obtain sufficient unambiguous long range NOEs to generate a structure. In particular, NOEs between the β-strands and the helix could not be unambiguously assigned. As our objective was to characterize the binding of the monomer, and not to determine the monomer structure per se, we generated a chemical shift-based structure.

**Figure 3:**
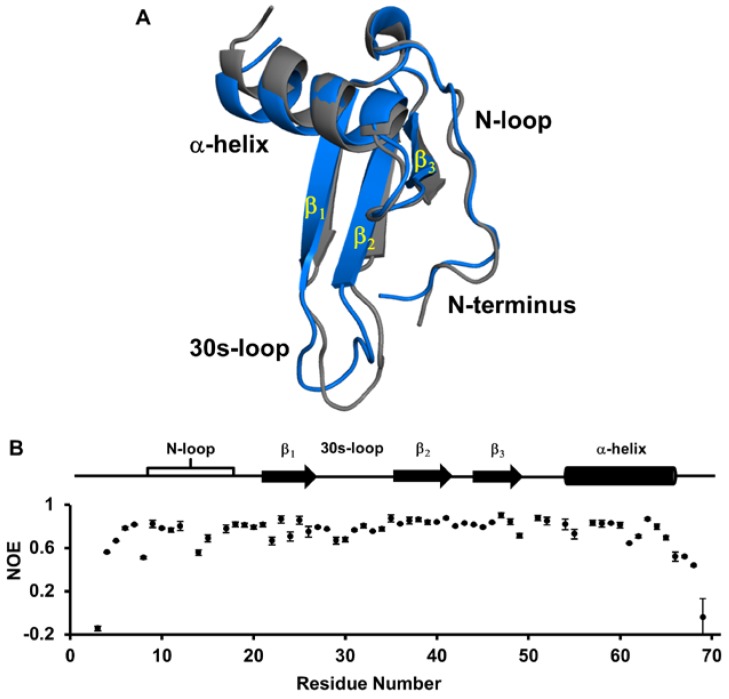
Structural features of the CXCL7 monomer. (**A**) Ribbon diagram of the CS-based CXCL7 monomer (blue) overlaid on a monomer of the tetramer (gray). Major structural regions are labeled; (**B**) Heteronuclear NOE data of the native CXCL7 monomer are shown. Secondary structural elements are given for reference.

It is now well established that ^1^H, ^15^N, Cα and Cβ chemical shifts can give an accurate structural model provided related structures are available. We first used our chemical shifts to predict the secondary structure and backbone torsion angles using TALOS-N [29,30]. Secondary structure prediction indicated three β-strands, an α-helix, as well as a structured N-loop commonly observed in chemokines. Predicted torsion angles were also well within favorable limits for a folded protein. We then generated a de novo monomer structure using CS-ROSETTA. The resulting structure was a well-folded protein with all the major chemokine structural motifs (Figure 3A). The torsion angles and intramolecular H-bonds were analyzed, and the torsion angles for 67 residues fall within favorable limits with the remaining three falling within acceptable limits.

We next compared our structure to the previously described monomer units of the tetramer crystal structure. Superimposition of the monomer units from the tetramer reveals a backbone root-mean-square deviation (RMSD) of 0.32 Å for structured β-strands and α-helix (Q20-G26, V34-L40 and R44-A64). Our structure showed a backbone RMSD of 0.82 Å compared to the monomer units of the tetramer over the same regions, and differences in these regions are mainly due to extended β-strands and a slight change in the orientation of the helix. The largest differences between our monomer and the tetramer structure were observed for the N-terminus and 30s-loop residues, which can be attributed to their conformational flexibility [16,31,32] (Figure 3A). In general, we observed that the more dynamic a region, the greater the difference in its backbone RMSD. We further examined the N-loop and helical regions as these are potentially involved in GAG and receptor interactions. Overall, the N-loop and helix closely resemble those of the crystal structure, with an average backbone RMSD of 0.64 Å. Our CS-based structure has a helix that spans from residues 54 to 64, similar to that observed in the tetramer. It is interesting that residues 65 to 70 are also unstructured in the tetramer, as C-terminal residues adopt a more defined helical structure in other NAC dimers. For instance, in CXCL8, the last six residues (66 to 72) are unstructured in the monomer, whereas the helix extends up to residue 70 in the dimer structures [19,22]. Similarly, only the last two or three residues are unstructured in the CXCL1 and CXCL5 dimer structures [13,18,23]. A shorter helix in CXCL7 could in part explain weak dimerization, as the corresponding residues in other NAC structures are involved in favorable interactions across the dimer interface.

To better understand the dynamic properties of the native monomer, we also carried out backbone ^1^H-^15^N-heteronuclear relaxation measurements. Heteronuclear NOEs are sensitive to motions in the picosecond-nanosecond timescale. Structured residues tend to have high NOE values (~0.8), and less structured or dynamic residues have lower NOE values. Our data indicated that the N-terminal residues preceding the CXC motif, C-terminal residues 66 to 70, and parts of the N-loop are dynamic, while the rest of the protein appears highly ordered (Figure 3B). Comparison of our data to the previously reported relaxation data of CXCL7 in the presence of chloroethanol shows striking differences for the 30s-loop residues. Heteronuclear NOE measurements in the presence of 2-chloroethanol indicate a highly dynamic 30s-loop, especially residues Q33, V34 and E35, showing very low NOE values observed for the very terminal residues, whereas our values are similar to those of structured residues [16,31,32]. These observations suggest that chloroethanol influences dynamic properties, and so, these data may not fully reflect the dynamics of the native protein.

### 2.3. CXCL7:CXCR2 N-Domain Interactions

Currently, nothing is known regarding the structural basis of how CXCL7 binds the CXCR2 receptor. Previous studies have indicated a two-site binding model for chemokine-receptor activation [33,34,35]. Site-I, which functions as a critical docking site, involves interactions between the chemokine N-loop region and receptor N-terminal domain. Site-II functions as the activating site and involves interactions between the chemokine N-terminal domain and receptor extracellular/transmembrane residues. As characterizing the structural basis of binding to the whole receptor is experimentally challenging, albeit possible [36], we used a divide and conquer approach to characterize the Site-I binding of CXCL7 to a CXCR2 N-terminal domain peptide. Such an approach has been extensively used to characterize Site-I interactions for a number of chemokines using different biophysical techniques including solution NMR spectroscopy [37,38,39,40,41,42,43,44,45].

**Figure 4:**
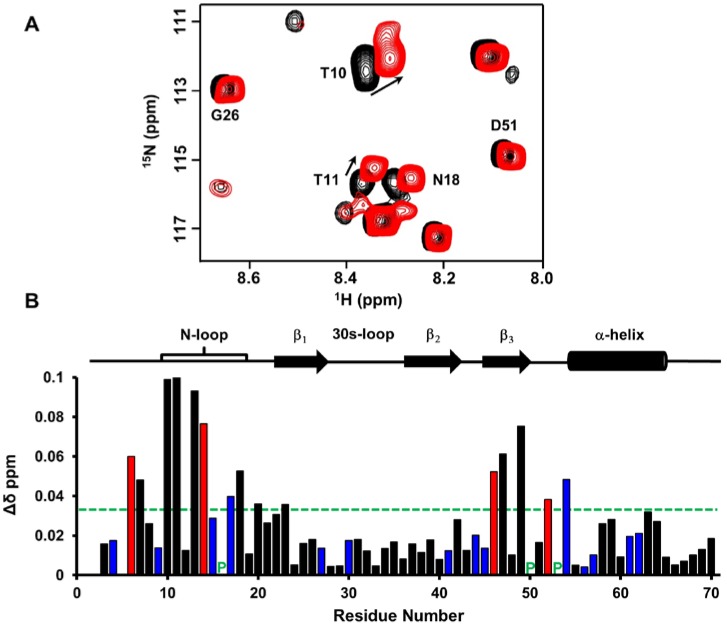
CXCL7 monomer binding to CXCR2 N-domain. (**A**) Portion of the 2D HSQC spectrum showing the overlay of CXCL7 in the free (back) and in the presence of CXCR2 N-domain at a 1:3.5 molar ratio (red). Residues showing significant perturbations are labeled and arrows indicate the direction of the peak movement; (**B**) Histogram plot of binding-induced chemical shift changes in the CXCL7 monomer as a function of amino acid sequence. Basic residues are shown in blue. Hydrophobic residues with significant CSP are shown in red. Prolines are indicated by a green ‘P’. Residues that show CSP above the threshold (dashed line) are considered involved in binding. Secondary structural elements are given for reference.

We characterized native chemokine monomer binding to the CXCR2 N-terminal domain (CXCR2Nd) at pH 6.0 using 2D-HSQC NMR titration experiments. Significant CSP was observed for hydrophobic residues M6, G13, I14, I46 and A52, polar residues C7, T10, T11, N18, Q20 and C47 and charged residues K17, E23, D49, R54 and the R44 side chain (Figure 4). Most of these residues constitute a continuous surface primarily along the N-loop and adjacent β-strand. The perturbation of cysteines is likely due to indirect interactions, as these residues are buried and so cannot be involved in direct interactions. Residues Q20 and E23 are located on the opposite face from the other residues, suggesting their perturbations are also due to indirect interactions.

**Figure 5:**
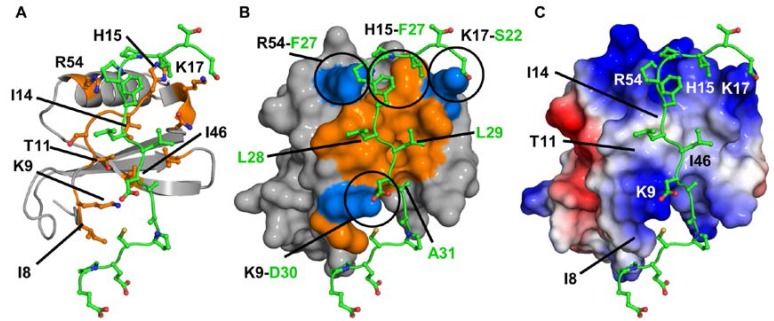
Model of the CXCL7-CXCR2 N-domain complex. (**A**) The ribbon diagram highlights the important binding residues on CXCL7 (orange). The receptor peptide is shown in green; (**B**) A surface filling model of the complex in the same orientation as shown in (**A**), highlighting residues involved in packing (orange) and ionic (blue) interactions. Several intermolecular interactions are circled and CXCL7 and receptor residues are labeled in black and green, respectively; (**C**) A schematic of the electrostatic surface in the same orientation as shown in (**A**,**B**) highlighting the hydrophobic pocket and the flanking basic residues. Important CXCL7 residues are labeled for reference.

To gain further insight into the binding, we generated a model for the CXCR2Nd-CXCL7 monomer complex using high ambiguity-driven biomolecular docking (HADDOCK)-based calculations that utilize CSP data as ambiguous interaction restraints along with shape complementarity and energetics to drive the docking process. Modeling revealed a single binding mode with the N-domain nestled along a groove between the N-loop and the β_3_-strand. Binding in our model is principally mediated by packing interactions between CXCL7 residues I8, T11, I14 and I46 and CXCR2 residues L28, L29 and A31 (Figure 5). Comparison of the chemokine sequences reveals that these residues are highly conserved (Figure 1), further indicating that they are critical to receptor binding. These observations also suggest that the CSP of M6, T10 and A52 are due to indirect interactions. In addition, we observe several transient interactions for charged and polar residues in many, but not all, of the models. These include an aromatic π-stacking interaction between CXCL7 H15 and CXCR2 F27 and an H-bonding interaction between CXCL7 K17 side chain NH_3_^+^ and CXCR2 S22 side chain hydroxyl groups. CXCL7 R54 is also involved in binding, forming either an H-bond between its guanidine side chain and CXCR2 P28 backbone carbonyl or a cation-π interaction with CXCR2 F27 (Figure 5). The remaining residues were not involved in direct binding interactions, indicating that their CSPs are likely due to binding-induced structural changes. Interestingly, H-bonding interactions were observed between the CXCL7 K9 side chain NH_3_^+^ and the CXCR2 D30 carboxylate, though K9 showed no chemical shift perturbation. It is likely that the absence of chemical shift changes is due to cancellation between contributions from direct and indirect interactions of similar magnitude but opposite sign. Lack of CSP of lysine residues involved in GAG binding has been previously observed in other chemokines [46,47,48,49]. Our docking model and CSP data collectively indicate that hydrophobic packing, guided by H-bonding and ionic interactions, mediates Site-I binding.

### 2.4. CXCL7 Monomer-Heparin dp8 Interactions

**Figure 6:**
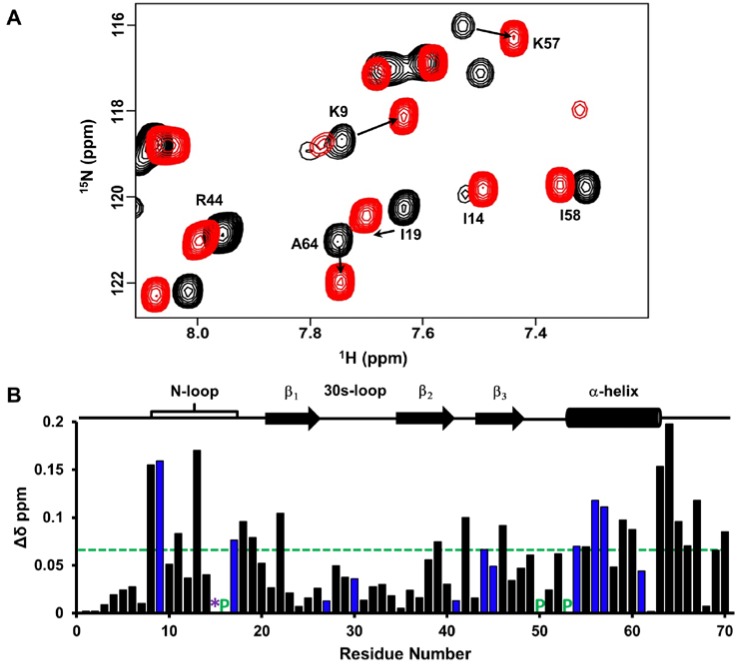
CXCL7 monomer binding to heparin dp8. (**A**) Portion of the HSQC spectrum showing the overlay of CXCL7 in the free (black) and in the presence of heparin dp8 at a 1:4 molar ratio (red). Residues that show significant perturbation are labeled; (**B**) Histogram plot of binding-induced chemical shift changes in CXCL7 monomer as a function of amino acid sequence. Residues that show CSP above the threshold (dashed line) are considered perturbed. Basic residues Arg, Lys and His are shown in blue. Residue H15 is broadened out in the free spectra and is represented by a “*”. Prolines are shown by a green “P”. Secondary structural elements are given for reference.

CSP analysis showed significant perturbation for residues in the N-loop, β_3_-strand and the α-helix (Figure 6). As basic residues are known to mediate GAG binding, we focused on residues K9, H15 and K17 from the N-loop, R44 from the β_3_-strand and R54, K56 and K57 from the helix. Peaks corresponding to residues H15 and K17 are broadened out in the free protein, but appear during the GAG titration, indicating that they are dynamic in the free form and become structured upon binding. We could measure the CSP for K17 as the peak appears early in the titration, but not for H15, as it appears late in the titration. CSPs of hydrophobic and acidic residues, which are located either proximal to basic residues or on the C-terminal helix (residues L63 to E67), are likely due to indirect interactions. Chemical shifts reveal that helical residues L63 to E67 are unstructured in the free form, and the observation that the shifts move upfield in the bound form suggests that GAG binding stabilizes and promotes the formation of the helix. Further, as we are able to simultaneously track the peaks corresponding to monomer and homodimer, we note that the equilibrium does not shift upon GAG binding and that monomer continues to dominate, indicating the monomer and dimer have similar affinities to heparin dp8.

**Figure 7:**
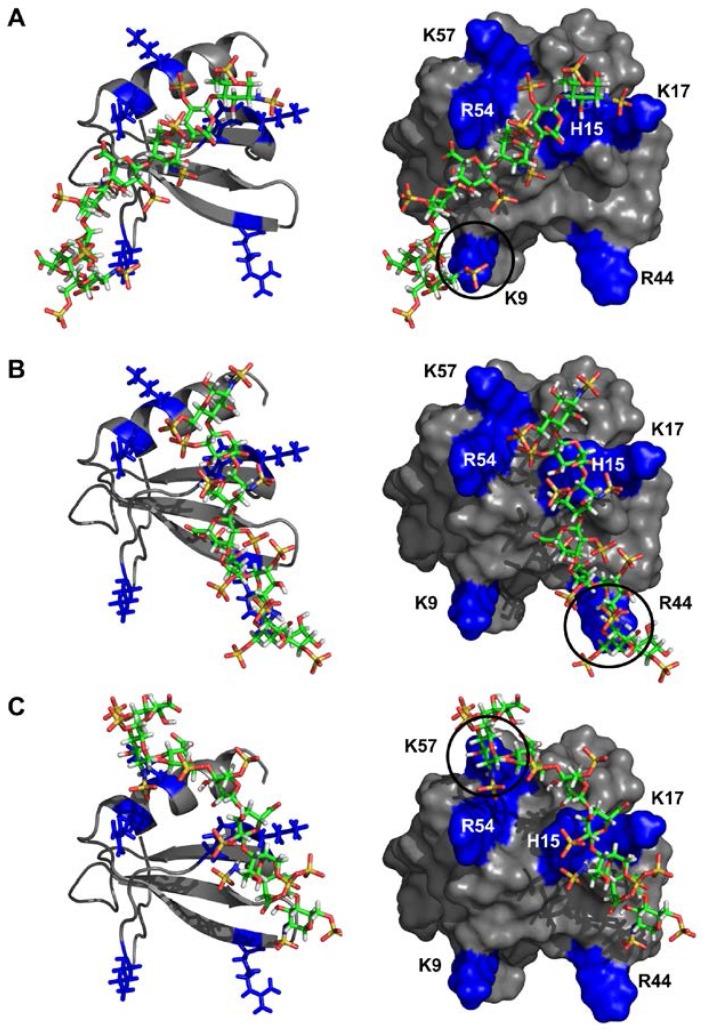
Models of the CXCL7-GAG heparin complexes. Different binding geometries that arise due to differences in peripheral interactions are shown in panels **A**, **B**, and **C,** respectively. The left column shows the ribbon diagram of the CXCL7 monomer, with GAG and positively-charged side chains shown as sticks. The right column shows the surface plots. Arg, Lys and His residues are highlighted in blue and labeled. Black circles highlight peripheral residues that mediate the binding geometry.

To gain insight into the binding geometry, we generated models of the dp8-bound structures using HADDOCK. All significantly perturbed residues, including hydrophobic and negatively charged residues, were used as restraints in generating the models. However, all of the models showed interactions with only basic residues indicating that the CSP of non-basic residues must be due to indirect interactions. Docking models resulted in several families, and interestingly, no one family could satisfy all of the residues that were perturbed in NMR CSP measurements. Models indicate that all binding geometries share a common core consisting of H15 and K17 of the N-loop and R54 of the α-helix. Whereas residues corresponding to H15 and K17 are highly conserved, R54 is unique and only present in CXCL7 (Figure 1). The GAG chain adopts three different orientations about this core due to selective binding to the peripheral residues K9, R44 or K57, defined as Models A, B and C, respectively (Figure 7). Structures failed to show interactions for K56, which is oriented away from the N-loop and towards the dimer interface, indicating that its CSP perturbations are due to indirect interactions. The same is true of the C-terminal residues L63 to E67, further supporting that their CSPs are due to structural changes. These data collectively indicate that the binding interface is plastic and that multiple binding geometries mediate monomer-GAG interactions. Additional docking experiments excluding one of the peripheral residues K9, R44 or K57 resulted in the two remaining geometries with no additional new geometries (Figure 7).

The only previous monomer-GAG characterization is for CXCL8 using an engineered monomer [46]. Interestingly, the binding interactions for the CXCL8 monomer were more stringent, with a single binding geometry similar to that observed in Model A. The more stringent geometry is mediated by a much larger core domain involving six residues in contrast to only three in CXCL7. Additional core residues in CXCL8 include the C-terminal helical residues R60, K64, K67 and R68 (corresponding to K57, K61, A64 and G65 in CXCL7). The smaller core in CXL7 appears to grant more degrees of freedom, allowing the GAG to adopt a range of geometries up to 180° about the core. Another key difference is K11 in CXCL8, the residue corresponding to K9 in CXCL7, which shows no interactions, and instead, K15 (equivalent to G13 in CXCL7), a residue unique to CXCL8, mediates binding. These data collectively indicate that both conserved and specific residues play differential roles in mediating GAG interactions and binding geometry in a chemokine-specific manner.

## 3. Discussion

CXCL7 plays a critical role in recruiting neutrophils to a variety of tissues, and dysregulation in this process has been implicated in inflammatory diseases, such as rheumatoid arthritis, acute lung injury and COPD [50,51,52], as well as a variety of cancers [53,54]. One of its primary functions involves neutrophil-platelet crosstalk during vascular injury, as it is released at relatively high concentrations from activated platelets and provides cues for directed neutrophil migration to the injury site [7]. However, nothing is known regarding the molecular level interactions between CXCL7 and its target receptor CXCR2 and GAGs.

As a member of the neutrophil activating chemokine family, CXCL7 shares several properties, such as a similar tertiary structure and activation of CXCR2 via the conserved ‘ELR’ motif. However, CXCL7 is unique, as it alone forms a weak dimer and also a tetramer at high concentrations, whereas other members form stronger dimers and no tetramers. Previous NMR studies have characterized binding interactions for native CXCL1, CXCL5 and CXCL8 dimers and engineered CXCL1 and CXCL8 monomers, as only dimers could be studied at concentrations used for NMR [37,39,46,48,49]. In this study, for the very first time, we have successfully characterized the binding interactions of an NAC monomer. By exploiting the weaker dimerization propensity and carefully varying solution conditions and protein concentration, we could assign the monomer chemical shifts that allowed mapping the binding interactions and generating structural models.

In addition to the ligand structure, knowledge of the receptor and ligand-receptor complex structures is also essential to fully describe residue-specific relationships between structural features, conformational changes and function. In recent years, structures of the free CXCR1 receptor and of other chemokine receptors bound with an antagonist and small molecule inhibitors have been reported [55,56]. However, structures of CXCR2 or the agonist-CXCR2 complex are not available. Therefore, our approach using the isolated N-domain and NMR chemical shift perturbation experiments can provide critical structural information that is otherwise difficult to obtain. Further, NMR CSP-based methods have been shown to be extremely useful for describing residue-specific GAG interactions, as protein-GAG complexes are notoriously difficult to crystallize. The CXCL7 monomer chemical shift assignments were previously reported in the presence of 2-chloroethanol [16,57]. Our chemical shifts were similar, but not identical, and interestingly, our heteronuclear relaxation data of the monomer showed a structured 30s-loop, whereas previous studies carried out in the presence of chloroethanol showed substantial dynamics similar to those observed for terminal residues. These observations highlight that dynamic studies carried out in the presence of reagents that disrupt native H-bonding interactions must be interpreted with caution.

Our NMR and modeling studies suggest that the N-loop and adjacent β-strand residues of CXCL7 mediate CXCR2 Site-I interactions. The binding mode and the nature of these interactions are similar to that observed for other CXCR2-activating chemokines CXCL1, CXCL5 and CXCL8 [37,39,49,58]. Binding is principally mediated by hydrophobic packing interactions that are conserved across the NAC family (Figure 1). However, charged residues unique to CXCL7, such as K9 and R54, also mediate binding, suggesting that such interactions fine-tune receptor activation and confer chemokine-specific function to what at first glance seems a redundant chemokine system. High resolution X-ray or NMR structures are essential to confirm and better describe the binding interactions at a single residue level.

**Figure 8:**
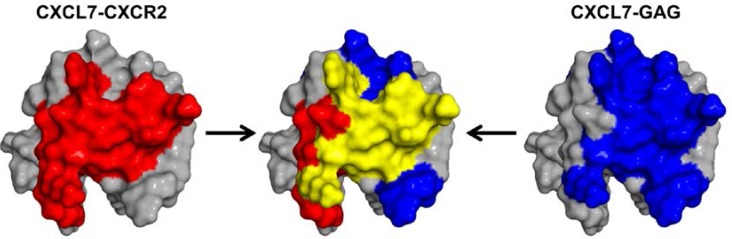
Overlap between GAG and CXCR2 binding domains. A schematic showing the CXCR2 binding domain (red), GAG binding domain (blue) and the overlap between the two domains (yellow).

Another important aspect of CXCL7 function is its interaction with GAGs. It is now well established that GAG binding plays a pivotal role in regulating chemokine signaling and establishing chemotactic/haptotactic gradients. Chemokines in solution form chemotactic and in GAG-bound form haptotactic gradients, but whether it is the GAG-bound or free chemokine that activates the receptor is not well understood. Our results indicate that GAG binding to the CXCL7 monomer is highly plastic and that GAG adopts multiple geometries. Independent of the binding models, a number of residues that mediate GAG interactions are also involved in receptor interactions indicating that the GAG-bound CXCL7 monomer cannot bind the receptor (Figure 8). Both monomer and dimer bound heparin dp8 with similar affinity and hence had no effect on the monomer-dimer equilibrium. However, though the dimer levels of the free chemokine are not dominant and the tetramer is favored at higher concentrations, the dimeric form, compared to the monomer, is favored on binding longer GAGs. During active neutrophil recruitment and tissue injury, it is very likely that local concentrations can vary by orders of magnitude, and so, it is possible that the levels of different oligomeric states and their GAG interactions are highly coupled and regulate in vivo function. We conclude that GAG interactions provide spatial and temporal control of receptor activity by modulating the amount of free chemokine and that the interplay between monomer-receptor and monomer-GAG plays an important role in mediating neutrophil recruitment in response to vascular injury.

## 4. Materials and Methods

### 4.1. Reagents and Protein Expression

CXCL7 was expressed in *Escherichia coli* cultured in either LB or ^15^N/^13^C enriched minimal medium and purified using a combination of nickel column and reverse phase high-performance liquid chromatography as described previously [44]. Purified protein was then lyophilized and stored at −20 °C until further use. The recombinant CXCR2 N-domain (residues 1 to 43) peptide was expressed using the same protocol as described above. Heparin dp8 oligosaccharide was purchased from Iduron. According to the manufacturer, the oligosaccharides were purified using high resolution gel filtration chromatography, consist mainly of the disaccharide unit IdoA,2S-GlcNS,6S (~75%), show some variation in sulfation pattern and contain uronic acid at the non-reducing end and a C4-C5 double bond as a result of the heparinase endolytic action.

### 4.2. Chemical Shift Assignments of the CXCL7 Monomer

NMR spectra were acquired using Bruker Avance III 600- and 800-MHz spectrometers equipped with cryoprobes and processed and analyzed using either Bruker Topspin 3.2 or Sparky programs [59]. Monomer chemical shift assignments were determined at 30 °C using a 300-µM protein sample in 50 mM phosphate, pH 4.0, containing 1 mM 2,2-dimethyl-2-silapentansesulfonic acid (DSS), 1 mM sodium azide and 10% D_2_O. The ^1^H and ^15^N chemical shifts were assigned using 3D ^1^H-^15^N heteronuclear NOESY and TOCSY experiments with mixing times of 150 and 80 ms, respectively. The carbon chemical shifts assigned from HNCA and CBCACONH experiments at pH 6.0 also helped in resolving some of the ambiguous assignments. The chemical shifts are shown in the Supplementary Material (Table S2). ^1^H-^15^N HSQC spectra collected from pH 4.0 to 7.5 in 0.5 increments were used to assign the backbone chemical shifts over this pH range.

### 4.3. NMR Titrations

Binding interactions of CXCR2 N-terminal domain and heparin dp8 to WT CXCL7 were characterized using solution NMR spectroscopy. A series of ^1^H-^15^N HSQC spectra were collected upon titrating either CXCR2 N-domain peptide or heparin to WT CXCL7 until no change in the chemical shifts were observed. The protein concentrations selected were high enough to obtain good quality spectra in a reasonable period. In the case of CXCR2 N-domain, we titrated 320 μM CXCR2 N-domain to 77 μM WT CXCL7 in 50 mM phosphate buffer at pH 6.0 and 35 °C. The final molar ratio of CXCL7:CXCR2 N-domain was 1:3.5. For CXCL7-GAG interactions, we titrated 10 mM heparin dp8 to a 50-μM sample in 50 mM phosphate pH 7.4 at 35 °C. The final molar ratio for CXCL7:octasaccharide was 1:4. For all titrations, chemical shift perturbations were calculated as a weighted average of changes in the ^1^H and ^15^N chemical shifts as described [58].

### 4.4. Model of the Monomer Structure

The monomer structure was generated using CS-ROSETTA, a robust tool for generating de novo structures from NMR chemical shifts [60,61]. The program uses the PDB database to select protein fragments based on the given backbone Cα, Cβ, N and NH chemical shifts and then assembles and relaxes these fragments into a converged structure using a ROSETTA Monte Carlo approach. Disulfide bonds were absent in the initial structure and subsequently added using PyMol. The structure was subjected to constrained energy minimization to allow the disulfides to adopt proper geometry, followed by global energy minimization and structural analysis using the AMBER 12 suite and VADAR [62,63].

### 4.5. Molecular Docking Using HADDOCK

Molecular docking of CXCR2Nd and heparin to the CXCL7 monomer was carried out using the high ambiguity-driven biomolecular docking (HADDOCK) approach as described previously [46,48,64,65]. The CXCL7 monomer structure determined using CS-Rosetta, the unstructured CXCR2 N-terminal peptide generated in Pymol and the NMR structure of heparin (PDB ID: 1HPN) [66] were used for docking. Ambiguous interaction restraints (AIRs) were selected based on NMR chemical shift perturbation results. The pair-wise “ligand interface RMSD matrix” over all structures was calculated, and the final structures were clustered using an RMSD cut-off value of 7.5 Å for CXCR2Nd and 4 Å for heparin. The clusters were then prioritized using RMSD and the “HADDOCK score” (weighted sum of a combination of energy terms).

## Supplementary Materials

Supplementary materials can be found at www.mdpi.com/1422-0067/18/3/508/s1.

## Abbreviations

CXCL	CXC ligand
CXCR2	CXC chemokine receptor 2
CXCL7/NAP-2	Neutrophil-activating peptide 2
GAG	Glycosaminoglycan
CXCR2Nd	CXCR2 N-terminal domain
GPCR	G-protein coupled receptor
NAC	Neutrophil activating chemokine
NMR	Nuclear magnetic resonance
HSQC	Heteronuclear single quantum coherence
CSP	Chemical shift perturbation
NOE	Nuclear Overhauser effect
AIR	Ambiguous interaction restraints
