# Supplementary material for: Structural Basis of Native CXCL7 Monomer Binding to CXCR2 Receptor N-Domain and Glycosaminoglycan Heparin
Source: Int J Mol Sci. 2017 Feb 26;18(3):508. doi: 10.3390/ijms18030508 (PMC5372524; doi:10.3390/ijms18030508)
Supplement: Supplementary file 1 [file ijms-18-00508-s001.docx]

Supplementary Materials: Structural basis of native CXCL7 monomer binding to CXCR2 receptor N-domain and glycosaminoglycan heparin

Aaron J. Brown, Krishna Mohan Sepuru, and Krishna Rajarathnam

**Table S1.** Distribution of CXCL7 monomers, dimers, and, tetramers as a function of solution conditions.

| **Concentration (µM)** | **pH** | **Temperature (**°**C)** | **Buffer** | **Distribution** |
| --- | --- | --- | --- | --- |
| 77 | 4.2 | 35 | 50 mM NaPi | ~95% M, ~5% D |
| 77 | 4.4 | 35 | 50 mM NaPi | ~85% M, ~15% D |
| 77 | 5.0 | 35 | 50 mM NaPi | ~80% M, ~20% D |
| 77 | 6.0 | 35 | 50 mM NaPi | ~70% M, ~30% D |
| 77 | 7.0 | 35 | 50 mM NaPi | ~65% M, ~35% D |
| 77 | 7.5 | 35 | 50 mM NaPi | ~60% M, ~40% D |
| 77 | 7.5 | 25 | 50 mM NaPi | ~65% M, ~35% D |
| 77 | 7.5 | 35 | 50 mM NaPi, 100 mM NaCl | ~65% M, ~35% D |
| 300 | 4.0 | 35 | 50 mM NaPi | ~95% M, ~5% D |
| 300 | 4.0 | 25 | 50 mM NaPi | ~95% M, ~5% D |
| 300 | 6.0 | 35 | 50 mM NaPi | ~50% M, ~50% D |
| 450 | 6.0 | 35 | 50 mM NaPi | ~55% D, ~45% M |
| 600 | 6.0 | 35 | 50 mM NaPi | M/D/T * |
| 840 | 6.0 | 35 | 50 mM NaPi | ~100% T |

M, D, T stand for monomer, dimer, and tetramer, respectively; Pi = phosphate. * relative ratios of M, D, and T could not be determined reliably.

**Table S2.** NMR assignments for CXCL7 monomer in 50 mM phosphate at pH 4.0 and 30 °C.

| **CXCL7 Sequence Assignments** | | | | | | | |
| --- | --- | --- | --- | --- | --- | --- | --- |
| Residue | NH | Hα | Hβ | others | N | Cα | Cβ |
| 1. Ala | - | - | - | - | - | - | - |
| 2. Glu | 8.66 | 4.43 | 2.02 | γH 2.39 | 120.63 | 53.71 | 28.84 |
| 3. Leu | 8.44 | 4.38 | 1.62 | γH 1.62, δH 0.84 | 124.47 | 52.45 | 43.93 |
| 4. Arg | 8.28 | 4.65 | 2.03, 1,81 | γH 1.59 δH 3.16 | 120.71 | 52.37 | 29.65 |
| 5. Cys | 8.36 | 4.65 | 3.50, 2.88 | - | 119.42 | 52.78 | 37.72 |
| 6. Met | 9.36 | 4.40 | 2.11 | γH 2.61 | 123.88 | 54.28 | 32.50 |
| 7. Cys | 8.41 | 4.90 | 3.06, 2.92 | - | 118.17 | 52.00 | 40.36 |
| 8. Ile | 8.44 | 4.19 | 1.98 | γH 1.27 δH 0.99 | 122.63 | 59.69 | 36.68 |
| 9. Lys | 7.91 | 4.66 | 1.94, 1.80 | γH 1.49 δH 2.93 | 119.97 | 52.50 | 33.22 |
| 10. Thr | 8.31 | 4.68 | 4.03 | γH 1.13 | 113.15 | 57.63 | 69.90 |
| 11. Thr | 8.38 | 4.73 | 4.05 | γH 1.23 | 115.77 | 58.73 | 69.04 |
| 12. Ser | 8.58 | 4.74 | 4.01 | - | 119.51 | 55.17 | 62.81 |
| 13. Gly | 8.58 | 3.99 | - | - | 110.36 | 43.87 | - |
| 14. Ile | 7.50 | 4.25 | 1.82 | γH 1.15 δH 0.79 | 118.94 | 56.11 | 36.70 |
| 15. His | 8.64 | 5.06 | 3.39, 3.21 | - | 124.66 | 52.32 | - |
| 16. Pro | - | - | - | - | - | 62.60 | 30.33 |
| 17. Lys | 8.65 | 4.27 | 1.90 | γH 1.46 | 116.99 | 55.63 | 29.81 |
| 18. Asn | 8.20 | 4.88 | 3.18, 2.86 | - | 115.28 | 50.73 | 37.99 |
| 19. Ile | 7.68 | 3.90 | 1.94 | γH 1.68 δH 0.76 | 120.19 | 60.38 | 37.21 |
| 20. Gln | 9.48 | 4.48 | 1.90 | γH 2.29 | 128.30 | 54.33 | 28.96 |
| 21. Ser | 7.94 | 4.68 | 3.90 | - | 111.31 | 54.98 | 63.60 |
| 22. Leu | 8.50 | 5.21 | 1.49 | δH 0.82 | 122.00 | 51.76 | 44.69 |
| 23. Glu | 8.85 | 4.72 | 2.06, 1.93 | γH 2.34 | 124.29 | 52.46 | 31.19 |
| 24. Val | 9.42 | 4.32 | 1.98 | γH 1.11 | 129.96 | 59.09 | 31.18 |
| 25. Ile | 8.67 | 4.72 | 2.00 | γH 1.50 δH 0.88 | 125.33 | 58.28 | 36.70 |
| 26. Gly | 8.59 | 4.14, 3.86 | - | - | 112.40 | 42.60 | - |
| 27. Lys | 8.22 | 4.16 | 1.77 | γH 1.25, δH 1.39 | 119.92 | 54.57 | 31.71 |
| 28. Gly | 8.41 | 4.35, 4.14 | - | - | 110.31 | 43.07 | - |
| 29. Thr | 8.35 | 4.68 | 4.07 | γH 1.09 | 114.23 | 62.27 | 67.05 |
| 30. His | 8.53 | 4.80 | 3.40, 3.17 | - | 115.71 | 53.94 | 28.80 |
| 31. Cys | 7.56 | 4.80 | 3.15 | - | 116.24 | 54.08 | 39.62 |
| 32. Asn | 8.70 | 4.87 | 2.96 | - | 126.38 | 51.17 | 36.25 |
| 33. Gln | 7.63 | 4.68 | 2.16, 1.92 | γH 2.37 | 116.81 | 51.49 | 41.14 |
| 34. Val | 8.34 | 4.18 | 1.94 | γH 1.08, 0.86 | 122.60 | 60.42 | 29.70 |
| 35. Glu | 8.71 | 4.80 | 2.02 | γH 2.55 | 124.20 | 53.19 | 30.80 |
| 36. Val | 9.47 | 4.82 | 2.11 | γH 0.84 | 124.05 | 57.93 | 30.96 |
| 37. Ile | 9.01 | 4.79 | 1.85 | γH 1.50, 1.17, δH 0.80 | 126.39 | 57.65 | 37.83 |
| 38. Ala | 9.63 | 5.27 | 1.30 | - | 132.89 | 47.47 | 19.16 |
| 39. Thr | 8.87 | 4.94 | 4.12 | γH 1.28 | 119.17 | 59.84 | 67.29 |
| 40. Leu | 9.48 | 5.09 | 2.27 | γH 1.75, δH 0.96 | 127.70 | 51.48 | 40.37 |
| 41. Lys | 8.48 | 4.03 | 1.86 | γH 1.47 | 120.96 | 56.90 | 30.51 |
| 42. Asp | 7.66 | 4.61 | 3.19, 2.65 | - | 114.81 | 50.73 | 38.60 |
| 43. Gly | 8.09 | 4.4, 3.58 | - | - | 108.32 | 42.33 | - |
| 44. Arg | 7.89 | 4.27 | 2.04 | γH 1.71 | 120.90 | 54.78 | 30.11 |
| 45. Lys | 8.19 | 5.53 | 1.72 | γH 1.50, εH 3.01 | 120.02 | 52.48 | 32.50 |
| 46. Ile | 9.01 | 4.70 | 1.80 | δH 0.84 | 119.35 | 56.44 | 40.30 |
| 47. Cys | 8.73 | 5.62 | 3.79, 3.31 | - | 121.81 | 54.10 | 44.73 |
| 48. Leu | 8.99 | 5.04 | 1.68 | γH 1.51, δH 0.80 | 123.31 | 49.91 | 42.12 |
| 49. Asp | 8.19 | 4.72 | 2.88, 2.60 | - | 121.85 | 48.97 | - |
| 50. Pro | - | - | - | - | - | 61.35 | 30.54 |
| 51. Asp | 8.08 | 4.70 | 2.90, 2.69 | - | 114.30 | 51.30 | 40.16 |
| 52. Ala | 7.50 | 4.64 | 1.59 | - | 126.18 | 48.14 | - |
| 53. Pro | - | - | - | - | - | 63.64 | 30.50 |
| 54. Arg | 8.78 | 4.14 | 2.02 | γH 1.82 | 115.40 | 56.51 | 28.31 |
| 55. Ile | 7.44 | 4.05 | 2.27 | γH 1.47, δH 0.82 | 117.01 | 58.36 | 40.44 |
| 56. Lys | 8.48 | 4.04 | 1.91 | γH 1.52 | 122.50 | 57.76 | 30.75 |
| 57. Lys | 7.52 | 4.13 | 1.91 | γH 1.62 | 115.92 | 56.49 | 30.59 |
| 58. Ile | 7.37 | 3.82 | 2.04 | γH 1.23, δH 0.86 | 120.17 | 62.12 | 40.45 |
| 59. Val | 8.33 | 3.50 | 2.27 | γH 0.99 | 120.61 | 64.22 | 30.07 |
| 60. Gln | 8.18 | 4.02 | 2.21 | γH 2.55 | 117.15 | 56.27 | 26.73 |
| 61. Lys | 7.68 | 4.18 | 2.21, 2.00 | γH 1.61 | 118.45 | 56.49 | 29.64 |
| 62. Lys | 8.11 | 4.24 | 1.98 | γH 1.60 | 118.95 | 55.43 | - |
| 63. Leu | 8.25 | 4.30 | 1.82 | γH 1.60, δH 0.88 | 118.99 | 53.93 | 39.86 |
| 64. Ala | 7.74 | 4.38 | 1.55 | - | 121.78 | 50.19 | 17.61 |
| 65. Gly | 8.02 | 4.04 | - | - | 106.93 | 43.17 |  |
| 66. Asp | 8.15 | 4.72 | 2.82 | - | 119.68 | 51.52 | 40.46 |
| 67. Glu | 8.39 | 4.45 | 2.26, 2.03 | γH 2.47 | 121.05 | 53.88 | 28.21 |
| 68. Ser | 8.26 | 4.45 | 3.95 | - | 116.34 | 56.06 | 62.72 |
| 69. Ala | 8.22 | 4.46 | 1.45 | - | 125.91 | 49.94 | 17.94 |
| 70. Asp | 7.96 | 4.53 | 2.79 | - | 122.99 | 53.41 | - |
| Side Chain | | - | - | - | N | - | - |
| 4. Arg | - | - | - | 7.19 | 121.22 | - | - |
| 18. Asn | - | - | - | 7.10, 7.67 | 112.30 | - | - |
| 20. Gln | - | - | - | 6.86, 7.40 | 111.85 | - | - |
| 32. Asn | - | - | - | 6.86, 7.55 | 111.30 | - | - |
| 33. Gln | - | - | - | 6.79, 7.48 | 112.00 | - | - |
| 44. Arg | - | - | - | 7.87 | 121.92 | - | - |
| 54. Arg | - | - | - | 7.35 | 120.75 | - | - |
| 60. Gln | - | - | - | 6.81, 7.44 | 111.20 | - | - |


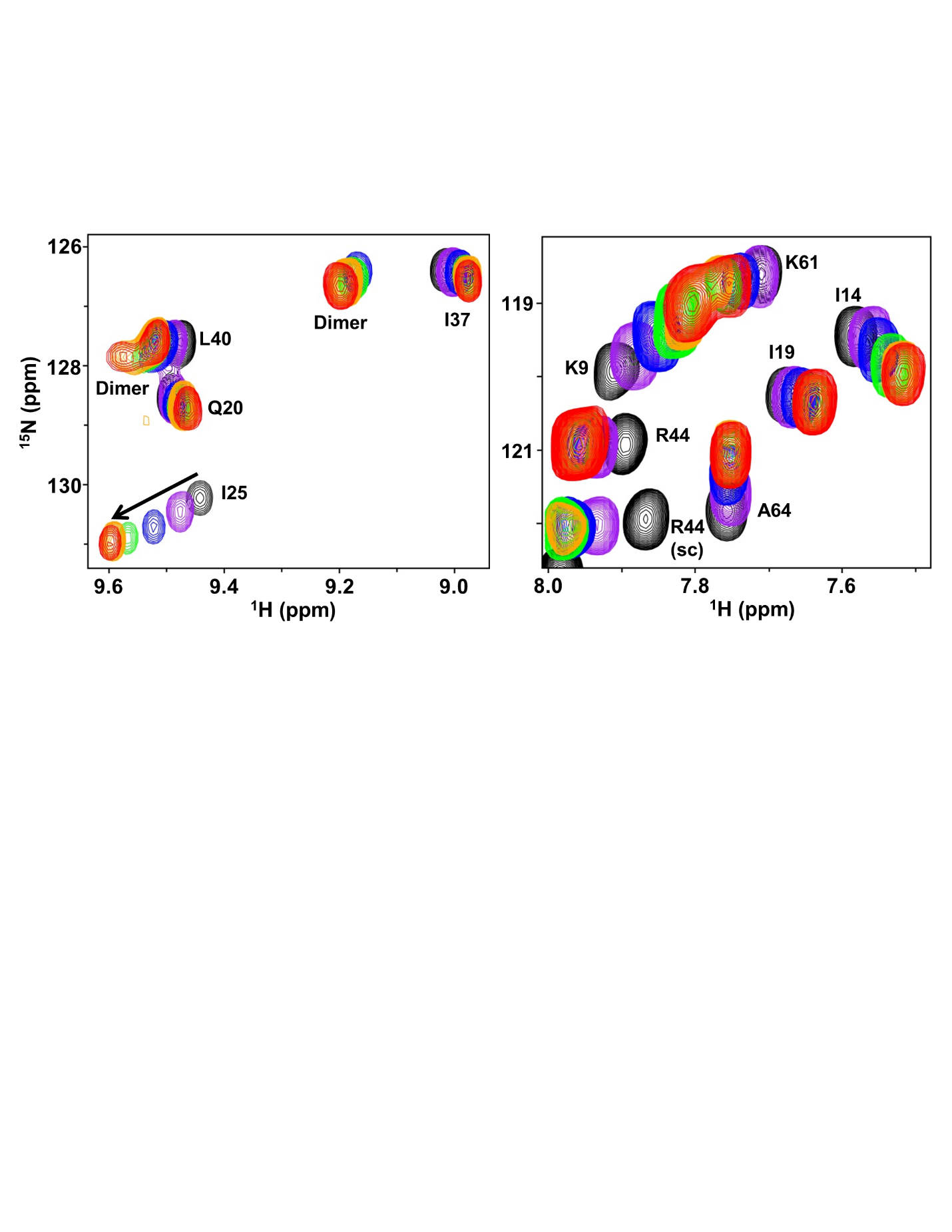


**Figure S1.** HSQC spectra of the pH titration from pH 4.0 to 7.0. Titration points go from pH 4.0 (black), 4.4 (purple), 5.0 (blue), 5.5 (green), 6.0 (orange), and pH 7.0 (red). Monomer peaks are labeled. Dimer peaks become visible at higher pH as indicated.
